# The Antimicrobial, Antioxidative, and Anti-Inflammatory Effects of Polycaprolactone/Gelatin Scaffolds Containing Chrysin for Regenerative Endodontic Purposes

**DOI:** 10.1155/2021/3828777

**Published:** 2021-09-30

**Authors:** Mahdieh Alipour, Bahareh Pouya, Zahra Aghazadeh, Hossein SamadiKafil, Marjan Ghorbani, Sara Alizadeh, Marziyeh Aghazadeh, Elaheh Dalir Abdolahinia

**Affiliations:** ^1^Dental and Periodontal Research Center, Faculty of Dentistry, Tabriz University of Medical Sciences, Tabriz, Iran; ^2^Faculty of Dentistry, Tabriz University of Medical Sciences, Tabriz, Iran; ^3^Stem Cell Research Center and Department of Oral Medicine, Faculty of Dentistry, Tabriz University of Medical Sciences, Tabriz, Iran; ^4^Drug Applied Research Center, Faculty of Medicine, Tabriz University of Medical Sciences, Tabriz, Iran; ^5^Stem Cell Research Center, Tabriz University of Medical Sciences, Tabriz, Iran; ^6^Research Center for Pharmaceutical Nanotechnology, Biomedicine Institute, Tabriz University of Medical Sciences, Tabriz, Iran

## Abstract

The appropriate endodontic material should eliminate the infection and inflammation to provide a situation for regeneration and healing of pulp tissue besides biomineralization. Chrysin is one of the active ingredients of plant flavonoids, which has significant anti-inflammatory and antimicrobial properties. In the present study, this natural substance was evaluated for antioxidant, anti-inflammatory, and mineralization properties on dental pulp stem cells (DPSCs). SEM, FTIR, and TGA tests were used to determine the successful synthesize of chrysin-loaded scaffolds. The antimicrobial effects of the synthesized scaffold against *Acinetobacter baumannii*, *Pseudomonas aeruginosa*, *Staphylococcus aureus*, and *Enterococcus faecalis* were assessed by the agar diffusion test and live/dead assay. The proliferation of DPSCs on these scaffolds was determined by the MTT assay, DAPI staining, and DNA extraction. Moreover, the antioxidant and anti-inflammation activity of chrysin-loaded scaffolds on inflamed DPSCs was evaluated. Alkaline phosphatase activity and Alizarin Red S Stain tests were done to evaluate the mineralization of DPSCs seeded on these scaffolds. The chrysin-loaded scaffolds reported antimicrobial effects against evaluated bacterial strains. The proliferation of DPSCs seeded on these scaffolds was increased significantly (*p* < 0.05). The TNF*α* and DCF levels in inflamed DPSCs showed a significant decrease in the presence of chrysin-loaded scaffolds (*p* < 0.05). The ALP activity and formation of mineralized nodules of DPSCs on these scaffolds were significantly increased compared with the control group (*p* < 0.05). These results indicated that chrysin as an ancient therapeutic agent can accelerate the healing and regeneration of damaged pulp tissue, and this active ingredient can be a potential natural substance for regenerative endodontic procedures.

## 1. Introduction

The dental pulp stem cells (DPSCs) are present in the living dental pulp and replaced injured odontoblasts, which is essential for the formation of reparative dentin after trauma, carious lesions, and other external stimuli [1, 2]. These progenitor stem cells are found in the dentin tissue as well as other connective tissues in the body and have a critical role in the repairing and healing of the inflamed and injured dentin-pulp complex [3].

Inflammation of the dentin-pulp complex usually occurs due to the infiltration of bacteria via dental caries [4, 5]. Lipopolysaccharides (LPSs) in the cell wall of gram-negative bacteria induce inflammation in pulp tissue [6]. LPS stimulates the production of blood cells (e.g., monocytes and lymphocytes) and proinflammatory fibroblasts which expressed cytokines (e.g., tumor necrosis factor (TNF) and Interleukin-1 (IL-1)) [7–9]. Increased levels of these proinflammatory cytokines stimulate different cell types to produce inflammatory mediators and chemokines to increase inflammatory responses, which can lead to pulp necrosis [10].

Flavonoids are part of the bioactive polyphenol family, which existed in the plant kingdom [11–15]. Propolis is a natural component that has been evaluated in various dentistry fields as a preventive agent against dental caries, a therapeutic agent for oral mucositis, plaque inhibitor, root canal irrigant, and pulp cap substance [16]. Moreover, this resinous substance with high polyphenols content has remarkable antimicrobial, antibiofilm, anticancer, antioxidant, and anti-inflammatory properties [17, 18].

Chrysin (5,7-dihydroxyflavone) is a natural flavonoid and is considered an active ingredient of honey, passion fruit, Indian trumpet flower, and European propolis [19–22]. Chrysin improves various health conditions such as hypertension, neurodegenerative disorders, ischemic retinopathy, diabetes diseases, coronary artery, and respiratory distress [20]. This active plant ingredient has various pharmacological benefits including anticancer, anti-inflammatory, antioxidant, antimutagenic, and antigenotoxic effects [23, 24]. Additionally, since in most cases, pulp tissue needs treatment due to the infection and the following inflammation, the antibacterial properties of the applied agents for regeneration of this tissue should provide notable antibacterial effects [25, 26]. It has been shown that the antimicrobial potential of chrysin compared with apigenin and lutein is higher against some strains such as *Escherichia coli*, *Salmonella typhimurium*, *Staphylococcus aureus*, and *Listeria monocytogenes* [27]. This characteristic has an essential role in the induction and progress of regenerative endodontics. Besides the antimicrobial and anti-inflammatory properties, this natural active ingredient induces osteogenic differentiation and mineralization [28, 29]. This property is essential for thickening dentinal walls, root development, and apical closure. Moreover, it is useful in dentin formation in direct and indirect pulp cap protocols related to mineralization [30, 31]. Among different active ingredients, the one chosen for endodontic regenerative material should provide a wide range of mentioned characteristics [32, 33].

Despite all the mentioned beneficial characteristics of chrysin, poor solubility in water, rapid intestinal and hepatic metabolism, low physicochemical characteristics, and poor stability are the major drawbacks of this substance limiting its application especially in load-bearing areas [24, 34]. To overcome these limitations, other polymers can combine with this active ingredient and improve the physicochemical properties. Different scaffolds were designed and fabricated for the loading of chrysin substance on them [23, 35, 36]. Polycaprolactone (PCL) is a semicrystalline biocompatible and biodegradable polyester, which is favorable for fabricating 3D fibrous scaffolds because of its notable hydrophobicity and high yield mechanical stability properties [37]. Moreover, gelatin is a natural polymer and one of the derivatives of collagen that could be an appropriate component of scaffolds due to low immunogenicity and similarity to the extracellular matrix (ECM) [38, 39]. Moreover, gelatin improves cell attachment and biodegradability of scaffolds and induces cell proliferation [40].

Due to the useful characteristics of chrysin in hard tissue regeneration such as its antimicrobial, anti-inflammatory effects, besides its mineralization potential, this study is aimed at evaluating the effect of this material on DPSCs as a potent regenerative endodontic biomaterial. In the current study, chrysin was loaded on polycaprolactone-gelatin scaffolds. The successful loading of this natural ingredient and toxicity of the fabricated scaffolds for DPSCs were assessed. Moreover, the antimicrobial activity, anti-oxidative, and anti-inflammatory effects of this scaffold were evaluated besides induction of mineralization in DPSCs.

## 2. Material and Methods

### 2.1. Fabrication and Evaluation of Chrysin-Loaded Scaffolds

1.2 g poly-ɛ-caprolactone (PCL) (Sigma-Aldrich Co.; Germany) polymer and gelatin (Merck; Germany) were dissolved in 12.5 mL dichloromethane (CH_2_CL_2_) (Merck; Germany) and water, respectively. Chrysin (97% purity, Sigma-Aldrich Co., Steinheim, Germany) was dissolved in the PCL solution (5% weight ratio). Then, two solutions with a ratio of 1 : 1 were mixed. In order to prevent the creation of a two-phase solution, polyvinyl alcohol (PVA) aqueous was used (1% volume ratio). The poly-ɛ-caprolactone-gelatin solutions without chrysin were mixed too. The mixtures were homogenized and freeze-dried for 48 hours at -20°C. Fourier transform infrared spectroscopy (FTIR) spectra of poly-ɛ-caprolactone-gelatin (PCL/Gel) and PCL/Gel/Chrysin samples in potassium bromide (KBr) tablets were carried out (Tensor27, Bruker, Germany). Interferograms were collected over the range of 500-4000 cm^−1^ with a minimal resolution of 2 cm^−1^ and 100 scans. Moreover, thermogravimetric analysis (TGA) (TGA/SDTA-851, Mettler Toledo, Spain) was fulfilled with a range of temperature (from 25°C to 600°C) at a heating rate of with a heating rate of 10°C per minute under nitrogen atmosphere. Morphology of developed scaffolds was showed using MIRA3 FEG-SEM (Tescan, Czech Republic). For this purpose, freeze-dried sample-covered gold was formed in a vacuum by sputter (SC7620-CF, Quorum Technologies, UK). Then, the images were observed by a KYKY-EM3200 microscope (Bio-equip, China) at an excitation voltage of 26 kV.

### 2.2. Antimicrobial Activity of Chrysin-Loaded Scaffolds

The antibacterial activity was done against four different strains including *Acinetobacter baumannii* (ATCC: BAA-747, Pasteur Institute, Iran), *Pseudomonas aeruginosa* (ATCC: 27853, Pasteur Institute, Iran), *Staphylococcus aureus* (ATCC: 6538, Pasteur Institute, Iran), and *Enterococcus faecalis* (ATCC: 13048, Pasteur Institute, Iran) using an agar well diffusion method. The strains were grown on Mueller Hinton Agar media (Sigma-Aldrich Co.), and 5 mm dimension circular samples (PCL/Gel and PCL/Gel/Chrysin) were placed in the center of plates. Incubation was performed at 37°C for 24 hours to measure the zone of inhibition surrounding each scaffold.

The viability of bacteria was determined using live/dead bacteria kit (LiFluor™ Bacteria Cell Viability Kit; Cat. #: C0028). Briefly, dentin discs with a radius of 4 to 5 mm and a thickness of 1 mm were prepared. NaOCl (1.5%) and EDTA 17% (pH 7.4) were used to wash the surface of the discs which were finally immersed in distilled water for 20 min. For sterile discs, the autoclave at 121°C was used for 20 minutes. Each of the discs is contaminated in a microtube containing BHI broth with 0.6% of bacterial suspensions. Samples are incubated at 37°C until 21 days at 100% moisture to facilitate bacterial biofilm formation. On day 21, the samples are gently washed with PBS to remove the culture medium and nonadherent bacteria. The specimens were then placed on the 24-well plates, and scaffolding was used to cover the dentin discs containing: PCL/Gel and PCL/Gel/Chrysin. After 7 days of incubation, the scaffolds were removed using 10 mL of distilled water. Samples stained using the Live/Dead Back Light Bacterial Survival Kit were imaged by Citation 5™. The percentage (%) of live bacteria was calculated using ImageJ analysis software (National Institute of Health, Bethesda, MD) by the ratio green fluorescence (live bacteria) to the total of the fluorescence intensities of red (dead bacteria) and green fluorescence multiple to 100.

### 2.3. Cell Viability of DPSCs Seeded on Chrysin-Loaded Scaffolds

Dental pulp stem cells were isolated and characterized as described before [41]. Briefly, these cells were isolated from extracted permanent teeth under local anesthesia due to orthodontic treatment in the Faculty of Dentistry, Tabriz University of Medical Sciences, after obtaining informed consent. After splitting the teeth, the pulp tissue was extracted and divided into small pieces and then digested in 3 mg/mL type I collagenase and 4 mg/mL dispase for 40 min at 37°C. The cells were cultured after centrifuging and incubated to reach 80% confluence. At the third passage, the cell suspension was analyzed by flow cytometry for the expression of surface markers including CD73, CD90, CD105, CD34, and CD45.

After 4 passages, 5000 cells of DPSCs were grown on each PCL/Gel or PCL/Gel/Chrysin scaffold placed into a 96-well plate and without scaffold as control. 200 *μ*L culture medium containing DMEM high glucose (Gibco Co.), 10% FBS (Gibco Co.), and 1X pen/strep (Gibco Co.) were added to each well, and the cell viability after 1, 3, and 5 days was measured by the MTT test. Briefly, 50 *μ*L of 3-(4,5-dimethylthiazol-2-yl-2,5-diphenyltetrazolium bromide) (Invitrogen, Carlsbad, CA, USA) solution (5 mg/mL) was replaced by 50 *μ*L of culture medium in each well and after 4-hour incubation at 37°C and 5% CO_2_. MTT/medium solution was replaced by 100 *μ*L DMSO. The absorbances of each sample were read at 570 nm by a multiwell plate reader (Bio Teck, Germany).

### 2.4. Evaluation of Cell Proliferation on the Scaffold

Samples, including PCL/Gel with/without chrysin and control, were stained by DAPI (4′,6-diamidino-2-phenylindole) (Cat. No. D9542, Sigma-Aldrich Co.) to assess the scaffold's effects on DPSC proliferation. The sterile scaffold was placed on 24-well culture plates. Then, the plates were seeded with DPSCs at a density of 5 × 10^4^ cells per well and cultured for 5 days. Before staining with DAPI, the samples washed with PBS (3x) were fixed using 4% paraformaldehyde for 10 min. After that, the samples washed with PBS (3x) were treated with Triton X-100 to improve cell permeability. Finally, the samples washed with PBS (3x) were stained with DAPI (3000 nm) (5 minutes). Samples were imaged via Cytation™ system (BioTek, Winooski, USA).

### 2.5. Measurement of DNA Content

DPSCs (cell density: cells per well) were seeded on 24-well plates in PCL/Gel scaffold with/without chrysin and control for 1, 3, and 5 days. The samples were then lysed before DNA extraction with Trizol reagent (Ambion Inc., Life Technologies, Carlsbad, USA) (0.3 mL/well). Chloroform (0.2 mL/well) was then used to separate the DNA from the organic and aqueous phases. Following centrifugation, the interphase phase of the samples (the DNA content) was extracted. The samples were suspended in 0.3 mL of 100% ethanol, 1 mL of sodium citrate (0.1 M), and 2 mL of 75% ethanol. The DNA pellet was then treated with NaOH (8 mM) to break down the cell membrane and extract the DNA. The materials were centrifuged at 12000 × g for 10 minutes to eliminate any insoluble debris. Finally, the supernatant was moved to a new tube, and the pH was set using a HEPES buffer. The DNA amount was measured spectrophotometrically using the NanoDrop 2000 instrument.

### 2.6. Anti-Inflammatory and Antioxidative Effects of Chrysin-Loaded Scaffolds

To evaluate the anti-inflammatory activity of fabricated scaffolds, the DPSCs were expanded on scaffolds with and without chrysin. These cells were stimulated with lipopolysaccharides (LPSs) of *E. coli* (Sigma-Aldrich Co., Steinem, Germany) to investigate the concentration of TNF *α* with and without chrysin-loaded scaffolds. LPS of *E. coli* is a principal component of gram-negative bacteria that activates the immune system. This part of bacterium is used for induction of inflammatory markers in culture medium in *in vitro* studies in which the researchers aimed to induce inflammation in dental pulp stem cells [42–44]. The production of TNF*α* was assessed using ELISA kits (DuoSet ELISA Development kit, Cat No. DY210-05).

ROS levels of DPSCs were evaluated by the DCFDA-Cellular Reactive Oxygen Species (ROS) Detection Assay Kit (Sigma-Aldrich Co., USA; Cat No. MAK142). The assay uses the cell-permeant reagent 2′,7′-dichlorofluorescein diacetate (DCFDA), a fluorogenic dye that assesses hydroxyl, peroxyl, and other ROS activities within the cells. After that, DCFDA is deacetylated by cellular esterases to a nonfluorescent compound, which is later oxidized by ROS into 2′,7′-dichlorofluorescein (DCF). This component is highly fluorescent that was detected by fluorescence spectroscopy.

### 2.7. Alkaline Phosphatase (ALP) Activity of DPSCs Seeded on Chrysin-Loaded Scaffolds

ALP activity was measured using p-nitrophenol phosphate as the assay substrate based on the manufacturer's instruction (Alp assay kit, Pars Azmoon, Iran). Briefly, 7 days after seeding of DPSCs on PCL/Gel and PCL/Gel/Chrysin scaffolds, the cells were lysed in alkaline lysis buffer. Samples were incubated at 37°C in a solution containing p-nitrophenol phosphate. The presence of p-nitrophenol was measured at an absorbance of 405 nm. The base of the experiment is

(1) $$\begin{array}{c} \text{p}‐\text{nitrophenyl phosphate}+{\text{H}}_{2}\text{O}\overset{ALP}{⟶}\text{phosphate}+\text{p}‐\text{itrophenol}. \end{array}$$

Total cellular protein was determined, and the ALP enzyme activity was normalized to total cellular protein.

### 2.8. Alizarin Red S (ARS) of DPSCs Seeded on Chrysin-Loaded Scaffolds

The ARS test was used to measure the calcium deposition on day 21 of culturing DPSC in differentiation medium, as described previously [45]. Briefly, the samples were washed by deionized water and then fixed with 2% paraformaldehyde (Sigma-Aldrich Co.) and stained with 40 mM Alizarin Red S solution (pH 4.2, Sigma-Aldrich Co.). After that, the wells were washed several times with water. In order to quantitative the results, ARS solution was extracted by adding a 10% acetic acid solution for 30 min with constant shaking and then neutralized with a 10% ammonium hydroxide solution, which was followed by colorimetric detection at 405 nm using an ELISA reader (Bio Teck, Germany).

### 2.9. Statistical Analysis

All analyses were carried out in triplicate, and data was reported as the mean ± SD. The results were analyzed by one-way ANOVA and Tukey's post hoc tests using Prism software (version 8.0, GraphPad, San Diego, CA, USA). *p* value < 0.05 was considered statistically significant.

## 3. Results

### 3.1. Fabrication and Characterization of Scaffolds

**Figure 1:**
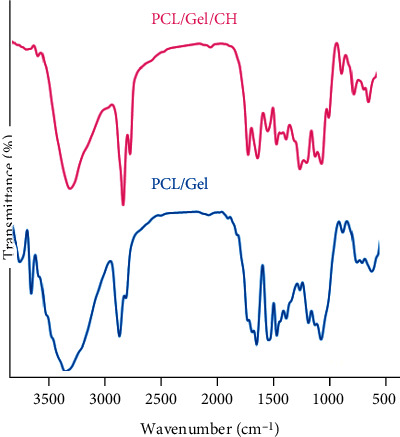
FTIR spectroscopy of PCL/Gel with and without Chrysin.

As demonstrated in Figure 1, the FTIR results of PCL/Gel showed the characteristic peaks at 1732 cm^−1^ (C=O stretching), 2925 and 2858 cm^−1^ (CH_2_ stretching), 1244 cm^−1^ (C-O and C-C stretching), and 1048 and 1165 cm^−1^ (C-O-C stretching). Moreover, the characteristic absorption peaks of gelatin were observed at 1643 cm^−1^ for C=O stretch of amide I, 1538 cm^−1^ for N-H bend of amide II, and 1372 cm^−1^ for amide III. Further peaks of gelatin were detected at 2948 cm^−1^, which was for aliphatic groups (-CH_2_ and -CH_3_), and 3429 cm^−1^ for O-H stretch, and 1460 cm^−1^ for -COO-. The spectrum of PCL/Gel/Chrysin offered a little shift in the position of characteristic bands due to the formation of hydrogen bonds between the chrysin and -OH or -NH_2_ groups in gelatin and PCL.

**Figure 2:**
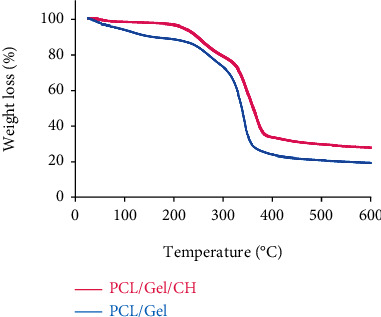
Thermogravimetric analysis (TGA) curves of PCL/Gel with and without Chrysin.

Thermal gravimetric analysis of PCL/Gel and PCL/Gel/Chrysin was performed to assess the thermal stability of developed scaffolds (Figure 2). As seen from TGA curves of PCL/Gel with and without Chrysin, the weight loss happened in 3 different stages: the first stage related to the evaporation of water which was below 200°C. The second stage displayed polymer thermal degradation from 200 to ~400°C, and the third stage from 400 to 600°C is attributed to the polymeric materials' carbonization. Moreover, weight of PCL/Gel with Chrysin was less than PCL/Gel, representing the chrysin content in the scaffold, which led to being more thermally stable thanks to the higher communications between chrysin and poly-ɛ-caprolactone-gelatin backbone.

**Figure 3:**
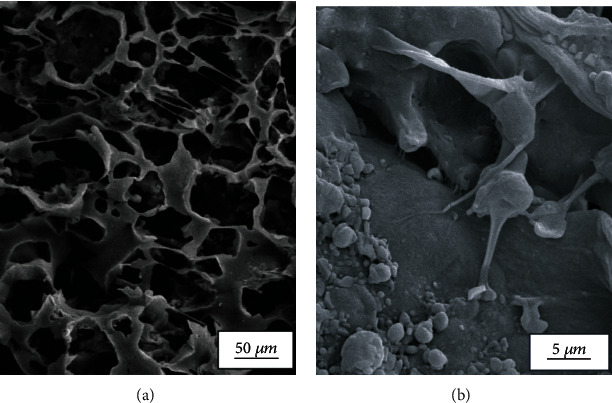
SEM images of (a) PCL/Gel/Chrysin scaffold and (b) the adhered and expanded DPSCs on CL/Gel/Chrysin on day 3 after the start of culture.

The inner microstructure of the lyophilized PCL/Gel/Chrysin with present and absent DPSCs was imaged by SEM. As shown in Figure 3(a), these freeze-dried scaffolds presented the morphology of the continuous polymer matrix. Morphology of DPSCs cultured on the surface of the developed scaffold was observed by SEM, too. As seen in Figure 3(b), these cells had good adherence and attachment to the scaffolds 3 days after seeding.

### 3.2. Antimicrobial Activity of Chrysin-Loaded Scaffolds

The antimicrobial abilities of PCL/Gel with and without Chrysin were assessed by the agar diffusion method, and the diameters of the zone of inhibition are shown in Table 1. The results confirmed the antimicrobial effects of loaded chrysin in scaffolds against *Acinetobacter baumannii*, *Pseudomonas aeruginosa*, *Staphylococcus aureus*, and *Enterococcus faecalis* strains (*p* < 0.01).

**Figure 4:**
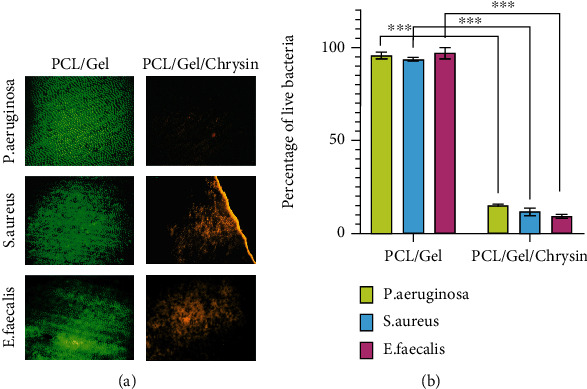
(a) Representative pictures of experimental groups showing live (green) and dead (red) cells of *E. faecalis*, *S. aureus*, and *P. aeruginosa* bacteria on dentin surface following PCL/Gel and PCL/Gel/Chrysin treatments. (b) Percentage of living bacterial cells after covering by PCL/Gel and PCL/Gel/Chrysin (*n* = 6) (^∗∗∗^*p* < 0.001).

Figure 4(a) depicts *E. faecalis*, *S. aureus*, and *P. aeruginosa* bacterial survivability on PCL/Gel and PCL/Gel/Chrysin, with green representing living bacteria and red indicating dead bacteria. According to Figure 4(b), the bacteria on the PCL were nearly entirely alive, showing that the PCL and gelatin in the scaffold lacked antibacterial characteristics consistent with prior studies [46–49]. In comparison, bacteria on PCL/Gel/Chrysin had a very poor bacterial population survival rate of less than 16% in all categories. Bacterial survival was significantly reduced on PCL/Gel/Chrysin as compared to PCL/Gel (*p* < 0.05). In the PCL/Gel/Chrysin groups, the rate of decreased bacterial growth was significantly higher in the *E. faecalis* group compared to the *P. aeruginosa* group (*p* < 0.05).

### 3.3. Viability of Seeded DPSCs on Chrysin-Loaded Scaffolds

**Figure 5:**
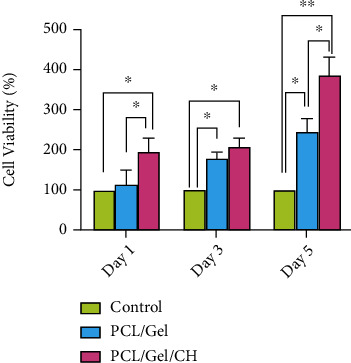
The viability of DPSCs seeded without scaffolds (control) and on the PCL/Gel with and without Chrysin. ^∗^*p* < 0.05 and ^∗∗^*p* < 0.01.

The cell viability of seeded DPSCs after 1, 3, and 5 days was compared with the control group. As demonstrated in Figure 5, the addition of chrysin to the structure of the scaffold significantly increased the cell viability compared to the control group. However, a significant difference between PCL/Gel with and without Chrysin was observed after 5 days.

### 3.4. Analysis of DNA Quantification and Visualization

**Figure 6:**
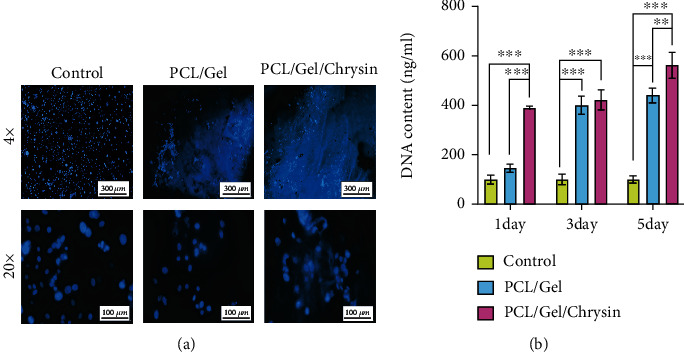
(a) The cell viability of the DPSCs by DAPI staining in control, PCL/Gel, and PCL/Gel/Chyrsin groups for 5 days. (b) The DNA content extracted from DPSCs grown into control, PCL/Gel, and PCL/Gel/Chrysin on days 1, 3, and 5.

To count nuclei, the cultured cells were stained with DAPI. Unexpectedly, the softness of the scaffold used in this study increases the possibility of cell penetration into the scaffold. As a result, no precise comparison of nuclei amount in cultured cells can be made between the control group and the cells grown on the scaffold (Figure 6(a)). Thus, DNA was extracted from the research groups to allow for a more accurate comparison. According to Figure 6(b), chrysin had the greatest effect on enhancing the total DNA content of DPSCs grown on scaffold compared to other groups on different testing days (*p* < 0.05). The DNA content recovered from PCL/Gel/Chrysin was about 4-5 times that of the control groups. There is no significant difference in DNA concentration among PCL/Gel/Chrysin samples on different days of culture. Cells grown on PCL/Gel on days 3 and 5 showed a significant increase compared to day 1. However, analyzing the DNA concentration of DPSCs on day 5 revealed that the PCL/Gel/Chrysin group had higher DNA content than the PCL/Gel group (Figure 6(b)).

### 3.5. Anti-Inflammatory and Antioxidant Effects of Chrysin-Loaded Scaffolds

**Figure 7:**
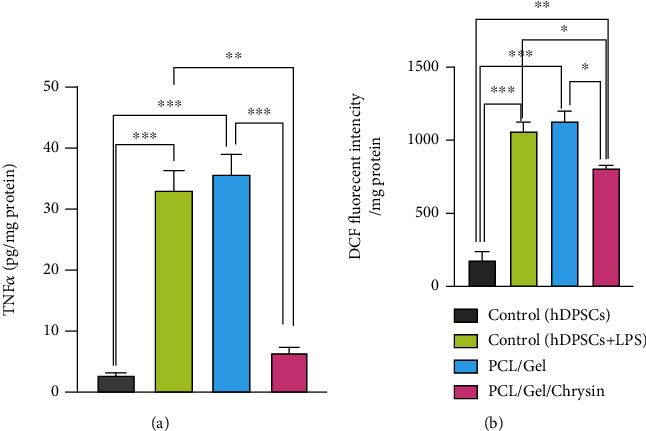
(a) Anti-inflammatory effects of PCL/Gel/Chrysin scaffolds on inflamed DPSCs induced by lipopolysaccharide (LPS). (b) Antioxidant effects of PCL/Gel/Chrysin on inflamed DPSCs induced by lipopolysaccharide (LPS). Data were reported as the mean ± SD (^∗^*p* < 0.05, ^∗∗^*p* < 0.01).

As shown in Figure 7(a), the concentration of TNF*α* was 2.68, 33.07, 35.535, and 6.405 pg/mg protein in control (DPSCs), control (DPSCs+LPS), PCL/Gel, and PCL/Gel/Chrysin groups, respectively. The increased levels of this proinflammatory cytokine were reported in the PCL/Gel. However, this increase was not statistically notable. The addition of chrysin in the scaffold remarkably decreased the levels of TNF*α*.

DCF levels were measured as antioxidant parameters, and the results were demonstrated in Figure 7(b). The significant decreases in DCF levels of inflamed DPSCs on chrysin-loaded scaffolds showed notable antioxidant effects of these scaffolds. They were in confirmation of the antioxidative effects of chrysin-loaded scaffolds.

### 3.6. Mineralization of DPSCs on PCL/Gel/Chrysin Scaffolds

**Figure 8:**
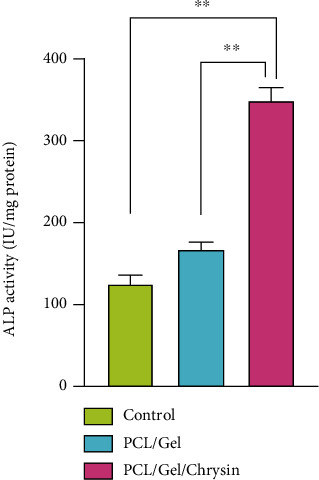
ALP enzyme activities after DPSCs were seeded on PCL/CS and PCL/CS/CH scaffolds after 7 days. Cells cultured without scaffold are considered control. Data reported as the mean ± SD performed in triplicate. The PCL/Gel/CH group showed significantly higher ALP activity compared to other groups (^∗∗^*p* < 0.01).

The ALP activity of DPSCs as determined in Figure 8 showed significant increases in the PCL/Gel/CH group after 7 days of cell culture (*p* < 0.05).

**Figure 9:**
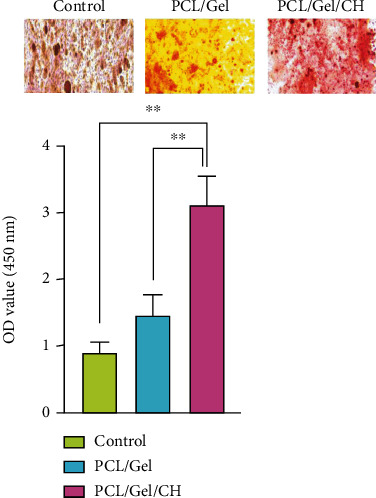
Alizarin Red staining of samples with the magnification of 200 *μ*m. Cells cultured without scaffold are considered control. The calcium content of the chrysin-loaded scaffolds was significantly higher than in other groups (^∗∗^*p* < 0.01).

The mineralization of DPSCs in these scaffolds is shown in Figure 9. Compared to the control group, DPSCs on PCL/Gel and PCL/Gel/CH showed higher ARS activity. Also, statistical increases in the formation of mineralized nodules were measured in chrysin-loaded scaffolds (^∗^*p* < 0.05).

## 4. Discussion

The aim of regenerative endodontic is to stimulate the regeneration of the damaged dentin-pulp complex. In dentin-pulp tissue, stimuli such as deep caries lead to differentiation of DPSCs to odontoblasts, which synthesize reparative dentin to preserve pulp vitality. However, the progression in carious lesions could lead to the failure of this defensive process [3]. The active ingredient of natural components is a promising substance for regenerative applications [13, 50, 51]. These active ingredients should be loaded on a suitable carrier for clinical applications. This carrier should have appropriate physical and chemical properties and be biocompatible and biodegradable. Poly(*ε*-caprolactone) (PCL) is a biodegradable and biocompatible polymer with various applications in regenerative medicine. This biopolymer has superior mechanical strength and could balance the solubility of natural components [52–54]. Gelatin is a natural polymer, which induces the colonization of DPSCs and revascularization of pulp tissue [55]. In the present study, chrysin was loaded in PCL/Gel scaffolds and freeze-dried to synthesize the scaffold for regenerative endodontic procedures. The SEM, FTIR, and TGA analyses were confirmed the successful fabrication of these scaffolds.

The leakage of bacteria and their by-products is the main cause of infection and inflammation in pulp tissue. The gram-negative bacteria have LPS in their cell walls that play a significant role in the inflammation of pulp tissue. *E. faecalis* is one of the main resistant species in infected root canals and failed root-filled canals [56, 57]. This strain is found in the saliva and root canal of patients with refractory apical periodontitis, which are resistant to treatments. Besides chemokine expression, which leads to inflammatory reactions, this bacterium inhibits osteoblast differentiation and Runx2 transcriptional activity. It is reported that *E. faecalis* reduces the expression of Runx2, osterix, and type I collagen genes and interferes with the hard tissue regeneration process [58, 59]. *P. aeruginosa* is another gram-negative bacteria that was founded in necrotic pulp and pulpitis [60]. A. baumannii and P. aeruginosa are other gram-negative and gram-positive bacteria in the infected pulp tissue, respectively [61]. Different studies were confirmed the antibacterial activity of propolis and its phenolic compounds. This natural agent can stimulate the immune system against bacteria or have bactericidal effects on them [62, 63]. The application of this substance as an intra-canal medicament significantly decreased the *E. faecalis* in infected root canals [64]. Moreover, the appropriate diffusion ability through infected dentinal tubules could increase the efficiency of this substance in long-term prognosis [65]. Chrysin is the main active ingredient in propolis. The presence of phenolic compounds in the chemical structure of chrysin is responsible for the antibacterial effects of propolis as well as other flavonoids [6, 62, 66]. In the current study, the significant antimicrobial effects of chrysin-loaded scaffold against four bacterial strains showed proved their efficacy in infected pulp tissue. We selected both common and important strains, which are known as critical causes in primary and refractory apical periodontitis.

The proliferative effects of propolis and its active ingredient on adipose-derived stem cells (ADSCs) and Stem Cells from Exfoliated Deciduous Teeth (SHED) were proved in previous studies [34, 67]. In the current study, the viability and proliferation of DPSCs on chrysin-loaded scaffolds were significantly increased in the presence of the active ingredient of propolis.

Hydrogen peroxide, hydroxyl radicals, and superoxide anion are common reactive oxygen species (ROS), which were increased around inflamed cells and tissues in the human body [68, 69]. The carious lesions cause some degenerative changes in the pulp tissue due to the increased levels of inflammatory cytokines and ROS. Propolis prevents inflammatory reaction besides disinfecting the infected pulp, which stops progressing to necrosis in the pulp tissue [63, 70]. The active ingredient of propolis in the current study was loaded in the polycaprolactone-gelatin backbone and evaluated on inflamed dental pulp stem cells. The results indicated the significant decreases in TNF*α* and DCF levels in chrysin-loaded scaffolds. However, the slight increases in levels of these markers were identified in inflamed DPSCs seeded on polycaprolactone-gelatin scaffolds. PCL is a nontoxic and safe polymer. However, the study conducted by Lee et al. exhibited mild inflammatory responses, which could relate to the accumulation of ROS surrounding the PCL-based scaffold [71]. Therefore, this mild increase in the PCL/Gel group could be in correlation to the presence of PCL polymer while the addition of chrysin in the PCL/Gel/Chrysin group significantly decreased the inflammation and oxidative stress in inflamed DPSCs.

Mineralization and formation of the continuous calcified barrier are important for the regeneration of the dentin-pulp complex especially in open apices [72].

During mineralization, calcium deposition occurs on the surface structure that is continued by the accumulation of phosphate ions, which turn the structure into the nucleation and growth sites of calcification. The red stains in ARS are formed due to the bonding of this agent to the calcium ions in calcified ECM [73]. Nucleation is considered the first stage of mineralization with a key role in calcification and mineralization. This procedure could accelerate the healing of injured periapical tissues and the induction of a mineralized barrier in open apex teeth [33]. ALP enzyme activity is another functional marker that is expressed during the nucleation procedure [74, 75]. The secretion of this enzyme in mineralized and calcified tissues such as the dentin-pulp complex is well established [76]. Generally, during dentinogenesis, undifferentiated dental pulp stem cells differentiate to odontoblasts that synthesize type 1 collagen and alkaline phosphatase [1].

In our study, the significant increases in ARS stained nodules were consistent with high ALP levels in the PCL/Gel/Chrysin. These results revealed the capacity of DPSCs to differentiate into various cells, which secrete mineralized matrix in the presence of chrysin. Therefore, these findings provide evidence to prove that chrysin as a natural active ingredient can promote the odontoblastic differentiation of DPSCs besides elimination of infection and inflammation, which make it an appropriate alternative for the regeneration of the dentin-pulp complex.

## 5. Conclusion

The ability to regenerate dentin-pulp complex with natural components would be a promising alternative in regenerative endodontic procedures. In the current study, the active ingredient of propolis was loaded in polycaprolactone-gelatin scaffolds. These scaffolds showed significant antimicrobial effects against bacterial strains found in the oral cavity. The cell viability of cultured DPSCs on these scaffolds significantly increased. Also, the anti-inflammatory and anti-oxidative capabilities of chrysin-based scaffolds were evidenced by the decreased levels of TNF*α* and DCF. Moreover, the loaded chrysin in a scaffold structure plays a key role as a bioactive agent with the ability to stimulate nucleation sites of mineralization besides enhancement of ALP activity. Thus, it has been concluded that these natural ingredient-based scaffolds could be attractive for the regeneration of damaged dentin-pulp complex.

## Figures and Tables

**Table 1:** Inhibition zone (mm) of PCL/Gel with and without chrysin.

Strain	Inhibition zone (mm)	
	PCL/Gel	PCL/Gel/Chrysin
*A. baumannii*	No detectable	8.5 ± 0.7
*P. aeruginosa*		7.5 ± 0.7
*S. aureus*		11 ± 1.41
*E. faecalis*		12 ± 2.82

## Data Availability

All data generated and/or analyzed during this study are included in this published article. The datasets used and/or analyzed during the current study are available from the corresponding author on reasonable request.
